# The Catheter Fell Out

**DOI:** 10.1016/j.atssr.2024.07.009

**Published:** 2024-07-26

**Authors:** Peyton Mashni, Clare Savage, Michael Jax, Anna Maria Reiter, Gordon Butler, Joseph B. Zwischenberger

**Affiliations:** 1 College of Medicine, University of Kentucky, Lexington, Kentucky; 2 Veterans Administration Hospital, Dallas, Texas; 3 Department of Surgery, University of Kentucky, Lexington, Kentucky; 4 College of Medicine, University of Texas Southwestern, Dallas, Texas

## Abstract

**Background:**

Accidental catheter removal or drain dislodgment, including tube thoracostomy, is a common, high-risk complication in hospitalized and ambulatory patients that often necessitates an additional procedure, increased length of stay, and increased cost.

**Methods:**

The aim of this study was to compare the tensile strength of pigtail catheter fixation using a simple interrupted suture, a U-stitch suture, or 2 simple interrupted skin sutures in a standardized skin model. Catheters were sutured to the skin, penetrating the collagen layer, with 1 of the 3 suture techniques and varying suture combinations.

**Results:**

For each trial, breakage occurred at the suture or knot. The mean breakpoint varied significantly between 2 simple interrupted sutures and both the 1 simple interrupted suture and the U-stitch technique (analysis of variance post hoc test *P* < .001), with the 2 simple interrupted suture technique withholding nearly 40% more force. Using the strongest suture, 0 silk, on a deceased adult sheep to secure a pigtail thoracostomy catheter yielded identical data compared with the standardized skin model.

**Conclusions:**

In conclusion, 2 simple interrupted skin sutures to secure a pigtail catheter has very low risk with a strongly positive benefit.

**In Short**

▪ In short, ACR is a common, high-risk complication in hospitalized and ambulatory patients that often necessitates an additional procedure, increased length of stay, and increased cost.
▪ This study compared the tensile strength of pigtail catheter fixation using a simple interrupted suture, a U-stitch suture , or 2 simple interrupted skin sutures in a standardized skin model.

Accidental catheter removal (ACR) or drain dislodgment is a common occurrence and a source of complications for patients in a variety of health care settings, with reports of 2.3 episodes per 1000 device-days.^1^, ^2^, ^3^, ^4^ Drainage catheters placed for fluid, air, blood, abscess, or bile require secure placement for days to weeks. In thoracic surgery, ACR of a tube thoracostomy or pigtail catheter can result in pneumothorax, hemothorax, or tension pneumothorax, with subsequent additional procedures, increased length of stay, increased cost, and potentially cardiac arrest. Hospitalized patients requiring the most complex level of care are also predisposed to ACRs during transport and health care team transfers. Additionally, this patient population often experiences confusion and delirium, thus further increasing the risk of ACRs. The risk of delirium dramatically increases with age, and older adults patients constitute a significant, increasing portion of the intensive care unit population.^5^

Whereas much literature exists comparing the superiority of suture materials and wound dressings, the number of knots when tying, or the number of “criss-crosses” or 360° wraps over the catheter, we evaluated the strength of suture-to-skin fixation. The aim of this study was to compare the tensile strength of catheter fixation with skin sutures in a standardized skin model using 3 techniques: a single simple interrupted suture (1S), a U-stitch suture (U), and 2 simple interrupted sutures (2S).

## Material and Methods

Using an anatomically correct skin model, a 12F pigtail catheter (Flexima nephrostomy catheter, Boston Scientific) was inserted percutaneously and secured with a 1S technique, a U technique, or a 2S technique. The skin model was composed of multiple layers of smooth-on silicone (dermal layers) poured onto 10% spandex mesh (collagen layer), one after the other to a desired thickness. Each layer was carefully measured and poured to meet the exact thickness of each of the layers of human skin. Each skin stitch, regardless of technique type, penetrated the epidermal and dermal layers to penetrate the dermal collagen analogue.

**Figure 1:**
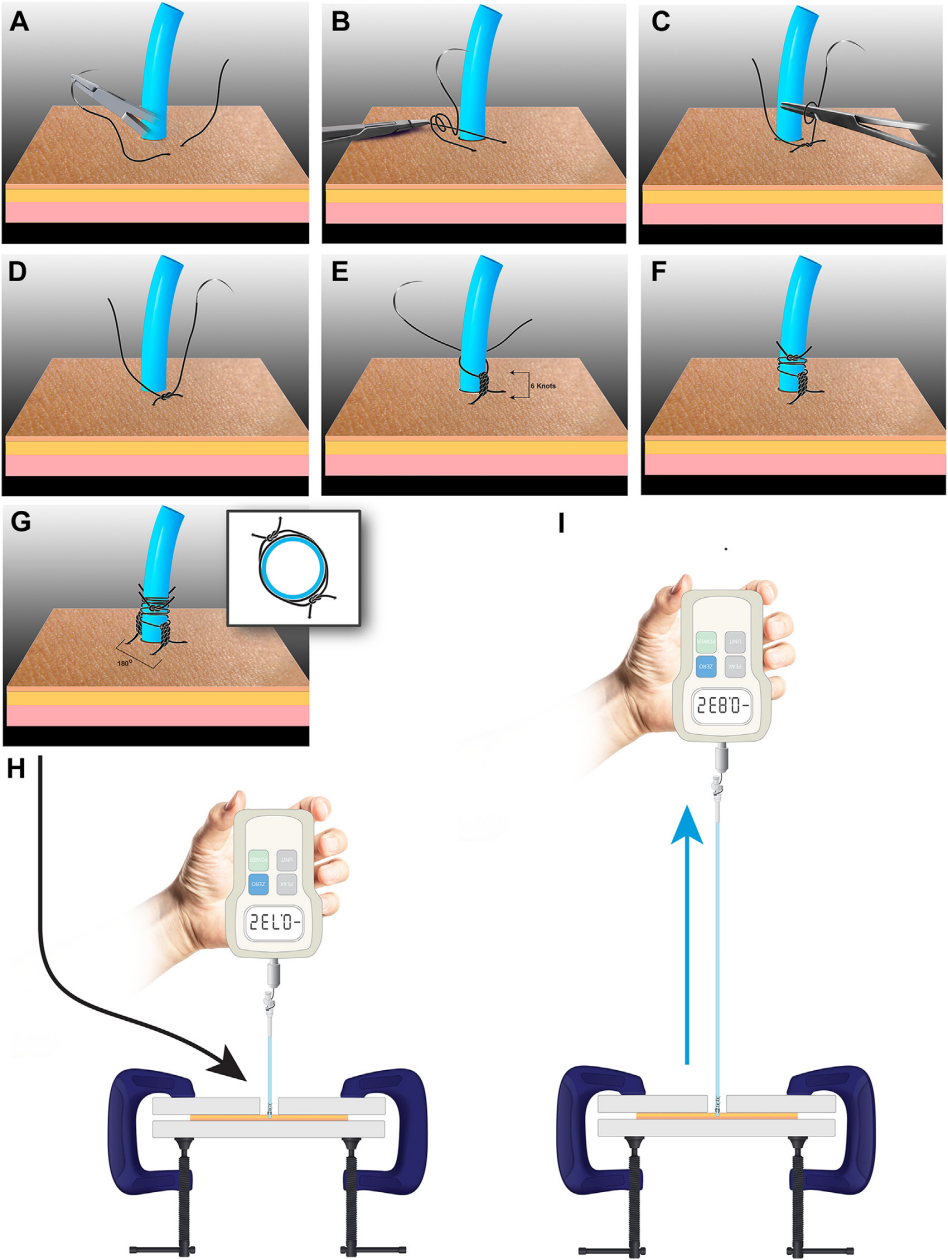
(A-I) Step-by-step illustration of the suture technique and force measurement setup.

The suture was secured to the skin with 7 knots to create a suture mesentery (Figure 1e). The suture was then tied to the catheter using two 360° wraps with 3 knots for each wrap. Suturing was performed by a single surgeon in a surgical simulation center to ensure proper, consistent technique. Once the sutures secured the catheter to the model skin, a wooden board with a round cutout for the catheter to be threaded through was placed on top of the skin and secured with 3 C-clamps, as seen in Figure 1.

An FGE-10XY Digital Force Gauge (Shimpo) was then hooked onto the catheter with a zip tie, and force was applied perpendicular (90°) to the skin. The breakpoint was recorded as the maximum tension force (kg) that the secured catheter-skin system was able to withstand before breakage. A total of 105 trials were recorded, 10 trials for each silk-technique combination (0 and 2-0, Ethicon Perma-hand Silk, Ethicon) and 5 trials for each monofilament-technique combination (0 and 2-0 polypropylene [Prolene, Ethicon] and 2-0 nylon [ Ethilon, Ethicon]).

This methodology was secondarily applied to a deceased adult sheep, which had been sacrificed on completion of a primary study. Because 0 silk was judged superior in the simulation studies, we repeated each step, securing an identical pigtail catheter inserted by percutaneous thoracostomy for a total of 10 trials using 5 1S technique sutures and 5 2S technique sutures. Each skin suture penetrated the sheep epidermal and dermal layers to penetrate the dermal collagen on the abdominal skin. All statistics were calculated using a 1-way analysis of variance and multivariate analysis of variance by a statistician using SPSS software (IBM Corp).

## Results

**Figure 2:**
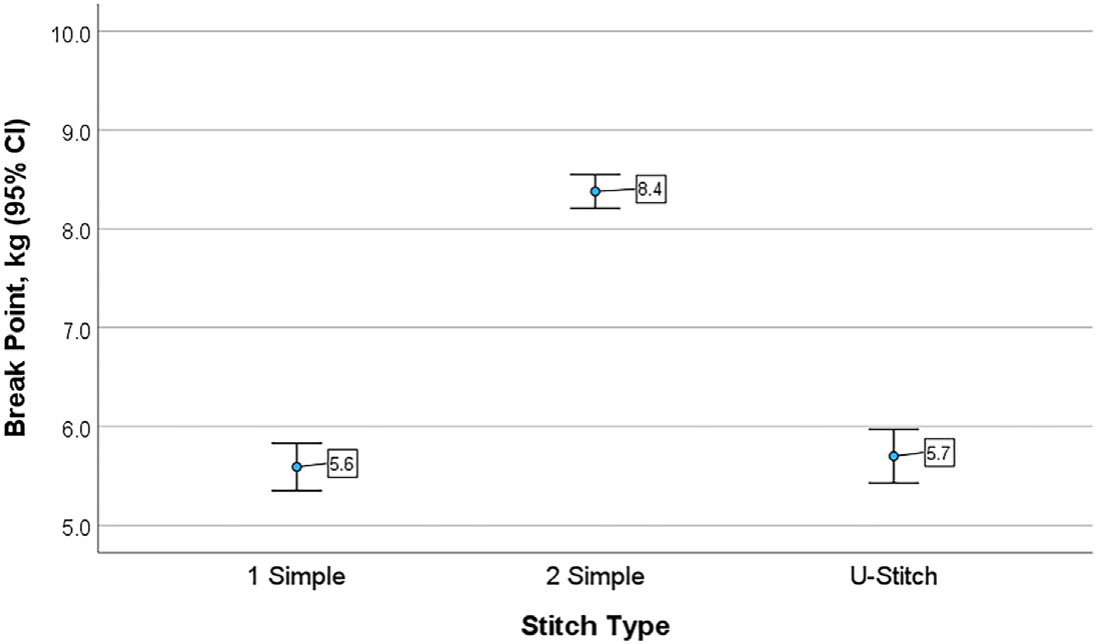
Mean breakpoint strength and SD in kilograms for 0 and 2-0 silk. Breakpoint varies significantly between the 2 simple interrupted sutures technique and the 1 simple or U-stitch technique (*P* < .001).

For each trial, breakage occurred at the suture or knot. No outliers were identified or excluded. The mean breakpoint for the 0 silk-1S suture combination was 5.994 ± 0.29 kg (Figure 2). The mean breakpoint using the 0 silk-2S suture combination was 8.308 ± 0.17 kg. The addition of 1 simple suture 180° apart yielded an average increase in breakpoint tensile strength of 39%. Similarly, the mean breakpoint for the 2-0 silk-1S suture combination was 5.185 ± 0.29 kg and for the 2-0 silk-2S suture combination was 7.234 ± 0.22 kg, with an average increase in breakpoint of 39%. The difference in mean breakpoint for the 1S and 2S techniques was statistically significant (*P* <.001) for both the 0 and 2-0 silk suture. No significant differences were noted between the U and 1S techniques (Figure 2, Table). The 0 silk served as the reference suture and was significantly stronger than either Prolene or Ethilon (Table).

**Table:** Multivariable Predictors From Linear Regression of Breakpoint Strength (kg) in an Anatomically Correct Skin Model^a^

Predictor	Regression Coefficient	95% CI	*P* Value
Suture size 2-0 vs 0	−.80	−1.07 to −.53	<.001
U-stitch suture vs 1 simple suture	.00	−.31 to .30	.978
2 simple sutures vs 1 simple suture	2.41	2.11 to 2.72	<.001
Ethilon (nylon, Ethicon) vs silk	−1.77	−2.16 to −1.37	<.001
Polypropylene (Prolene, Ethicon) vs silk	−1.08	−1.37 to −.79	<.001

a The baseline strength at 0 silk suture with 1 simple interrupted suture was 6.15 kg (95% CI, 5.88-6.43).

**Figure 3:**
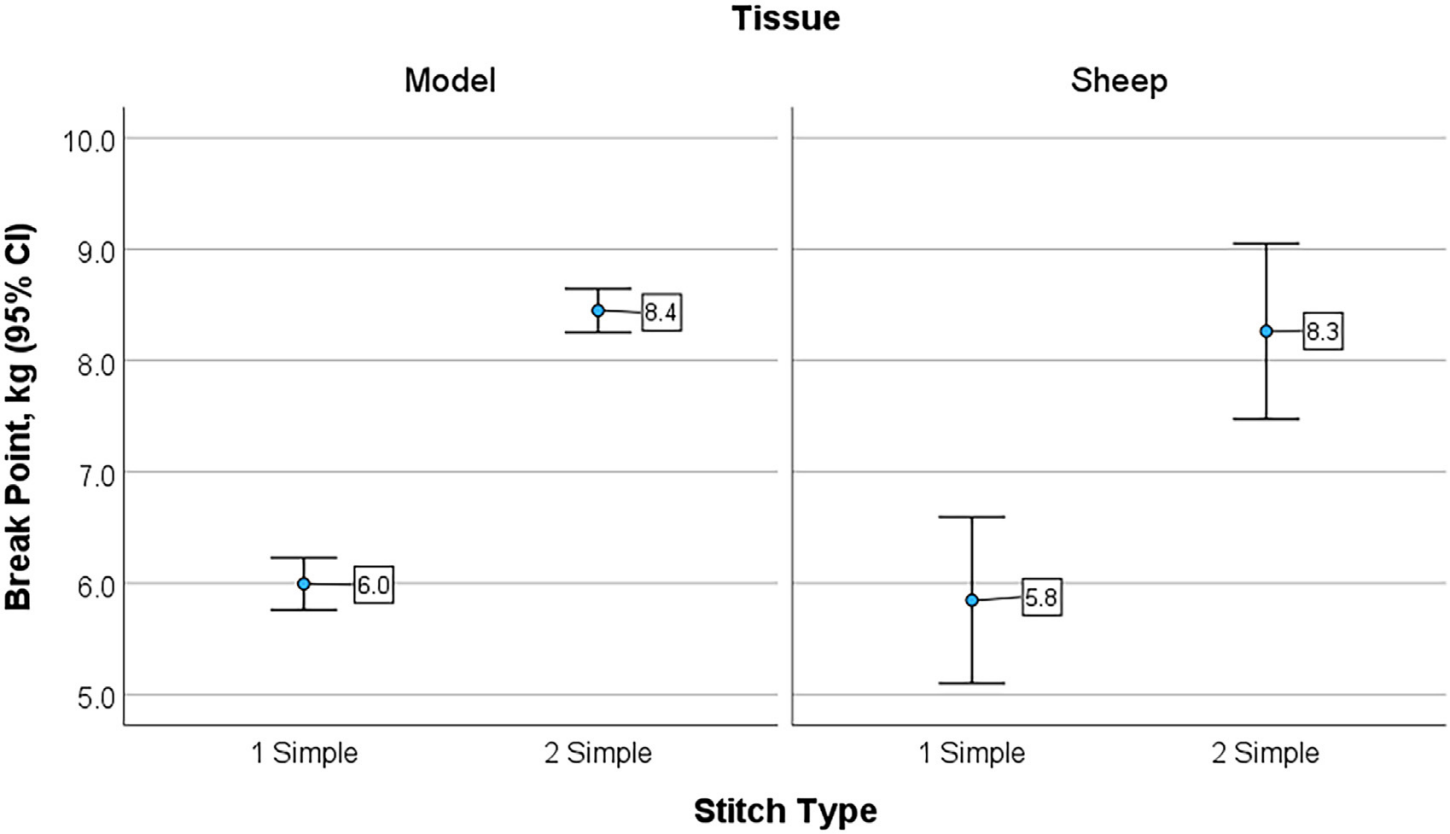
Mean breakpoint strength and SD in kilograms for the 1 simple interrupted suture technique and the 2 simple interrupted sutures technique in both the standardized skin model and sheep skin.

The results on the deceased adult sheep using 0 silk (reference suture) for the 1S and 2S techniques yielded nearly identical data compared with the standardized skin model, with the 2S technique stronger than the 1S technique (*P* < .001) (Figure 3). The sheep skin was similar to the standardized skin model, with a mean breakpoint of 0.17 kg less (*P* = .278).

## Comment

These data suggest that the breakpoint of a skin-affixed catheter system increases by almost 40% (3 kg of force) when an additional suture is placed through the skin, regardless of the suture material used. Specifically, the 2S technique is significantly stronger than either the 1S technique or the U technique, both of which are roughly equivalent in strength. No statistically significant differences were noted between the U and 1S techniques, thus suggesting that 1 single breakpoint is the culprit for the drain insecurity. The suture was secured to the skin with 7 knots to create a suture mesentery that limits mobility of the catheter and serves as a spring to absorb shock during sudden movements. The break strength of the suture materials tested is also confirmed as silk > Prolene > Ethilon. Similarly, 0 silk is stronger than 2-0 silk, although suture material was not the primary end point of the study.

Currently, the literature on catheter fixation technique does not discuss the number of skin sutures as a factor contributing to durability or as a technique to reduce the incidence of ACRs.^1^, ^2^, ^3^, ^4^ The lack of attention to the number of sutures placed may reflect expedience or fundamental dogma related to minimizing surgical site infections (SSIs) because every trauma and foreign material poses a potential nidus for skin infection. Most catheters that are affixed with skin sutures, however, can be considered class 1 wounds when securing a drainage catheter.^6^ These wounds should be classified as “clean” wounds subject to the low, widely accepted reported SSI rate of 1% to 5%. The risk and cost of SSIs in a superficial skin suture cannot be compared with the risk of losing a catheter placed to address a complex or life-threatening problem or with the risk and cost of subsequent morbidity or mortality. Another factor that may contribute to the preference for placing only a single suture is the assumed short timeframe for the drain to remain in place. However, many drains remain in place longer than planned, and the minimal additional time to better secure the drain can be crucial in ensuring that the drain remains secured to the skin.

Within each group using the standardized skin model, the breakpoint data were very consistent, with little variability. Similarly, repeating the 0-silk data on a fresh cadaver sheep validated the skin model for assessing catheter fixation. Suture depth as a variable was controlled in this study to include the model collagen layer, but in practice the suture must penetrate the skin collagen layer to duplicate the results. The selection of a lateral chest wall thoracostomy was arbitrary; perhaps the back (dorsal) skin of the sheep would have been stronger, but that would not influence the outcomes determined by suture breakage. As noted previously, force was applied to the skin at 90°. Additional force vectors were beyond the scope of this study.

Despite the consistent data in our skin model and cadaver sheep model, human clinical trials will be required to evaluate the methodology further. Finally, one could assume that additional sutures (3 or 4) would improve on the results of our study, but that is beyond our scope and of questionable clinical relevance. Finally, many catheters are secured with the patient under sedation. Once alert, the patient may become ambulatory, confused, or combative. Patient transport, visitors, nurses, and rehabilitation activity efforts all contribute to “the tube falling out.”

In conclusion, regarding the risk or benefit of adding a second suture to secure a tube thoracostomy pigtail catheter, the risk is low, but the benefit is significant.
